# Cumulative Effect and Predictive Value of Genetic Variants Associated with Type 2 Diabetes in Han Chinese: A Case-Control Study

**DOI:** 10.1371/journal.pone.0116537

**Published:** 2015-01-14

**Authors:** Yun Qian, Feng Lu, Meihua Dong, Yudi Lin, Huizhang Li, Juncheng Dai, Guangfu Jin, Zhibin Hu, Hongbing Shen

**Affiliations:** 1 Department of Chronic Non-communicable Disease Control, Wuxi Center for Disease Control and Prevention, Wuxi 214023, China; 2 Department of Epidemiology and Biostatistics, School of Public Health, Nanjing Medical University, Nanjing 210029, China; Tulane School of Public Health and Tropical Medicine, UNITED STATES

## Abstract

**Background:**

Genome-wide association studies (GWAS) have identified dozens of single nucleotide polymorphisms (SNPs) associated with type 2 diabetes risk. We have previously confirmed the associations of genetic variants in *HHEX, CDKAL1, VEGFA* and FTO with type 2 diabetes in Han Chinese. However, the cumulative effect and predictive value of these GWAS identified SNPs on the risk of type 2 diabetes in Han Chinese are largely unknown.

**Methodology/Principal Findings:**

We conducted a two-stage case-control study consisting of 2,925 cases and 3,281controls to examine the association of 30 SNPs identified by GWAS with type 2 diabetes in Han Chinese. Significant associations were found for proxy SNPs at *KCNQ1* [odds ratio (OR) = 1.41, *P* = 9.91 × 10–16 for rs2237897], *CDKN2A/CDKN2B* (OR = 1.30, *P* = 1.34 × 10–10 for rs10811661), *CENTD2* (OR = 1.28, *P* = 9.88 × 10-4 for rs1552224) and *SLC30A8* (OR = 1.19, *P* = 1.43 × 10-5 for rs13266634). We further evaluated the cumulative effect on type 2 diabetes of these 4 SNPs, in combination with 5 SNPs at *HHEX, CDKAL1, VEGFA* and *FTO* reported previously. Individuals carrying 12 or more risk alleles had a nearly 4-fold increased risk for developing type 2 diabetes compared with those carrying less than 6 risk alleles [adjusted OR = 3.68, 95% confidence interval (CI): 2.76–4.91]. Adding the genetic factors to clinical factors slightly improved the prediction of type 2 diabetes, with the area under the receiver operating characteristic curve increasing from 0.76 to 0.78. However, the difference was statistically significant (*P* < 0.0001).

**Conclusions/Significance:**

We confirmed associations of SNPs in *KCNQ1, CDKN2A/CDKN2B, CENTD2* and *SLC30A8* with type 2 diabetes in Han Chinese. The utilization of genetic information may improve the accuracy of risk prediction in combination with clinical characteristics for type 2 diabetes.

## Introduction

Type 2 diabetes is a major health problem that affects more than 300 million individuals worldwide [1], and its prevalence is continuously increasing in many countries, especially in China [2]. Genetic factors contribute to the pathogenesis of type 2 diabetes. Identifying its relevant genetic variants is critical in the risk prediction and targeting of preventive interventions for type 2 diabetes. Success in identifying type 2 diabetes associated genetic variants leads to suggestions that they may be useful in predicting an individual’s risk of developing the disease.

Genome-wide association studies (GWAS) have made great advances in identifying susceptibility genes associated with type 2 diabetes. Since the first GWAS reported four new genes for type 2 diabetes risk in 2007, there have been dozens of genes discovered through GWAS [3–19]. As most of these genes have been identified in populations of European ancestry, it is necessary to evaluate the relevance of these genes in non-European populations. In the Chinese population, we and others have replicated the initial single nucleotide polymorphisms (SNPs) indentified from the first wave of type 2 diabetes GWAS [20–31]. However, recently reported SNPs in European populations have not yet been systematically evaluated in the Chinese population. Research conducted in French individuals suggested that a reliable discriminating power to identify subjects with high susceptibility for type 2 diabetes could be reached with 15 SNPs [32].

Here, we tested the association of 39 SNPs from 30 genes GWAS identified with type 2 diabetes in a two-stage case-control study consisting of 2,925 cases and 3,281 controls in Han Chinese. We further evaluated the joint effect of related genetic variants and the performance of these SNPs on risk prediction for type 2 diabetes.

## Methods

### Ethics statement

Written informed consent was obtained from every participant and this study was approved by the Ethical Committee of Nanjing Medical University.

### Study subjects

The study was a two-stage (i.e. the discovery stage and the replication stage) case-control study and a total of 6,206 participants were included, which has been previously described [28]. All participants were derived from two community-based cross –sectional surveys. Subjects were considered to be type 2 diabetes cases if they had a history of type 2 diabetes or if their fasting blood glucose (FBG) was ≥ 7.0 mmol/l. Those without history of diabetes, hypertension, coronary heart disease, stroke, cancer and with FBG < 5.6 mmol/l were selected as controls. The discovery stage contained 1,200 type 2 diabetes cases and 1,200 controls while the replication stage contained 1,725 type 2 diabetes cases and 2,081 controls. All participants were unrelated and of Chinese Han ancestry residing in Jiangsu Province, China.

### SNPs selection and genotyping

According to reported 76 SNPs of type 2 diabetes from GWAS [3–19] through the GWAS database of National Human Genome Research Institute, we excluded 15 SNPs which had minor allele frequency (MAF) < 0.05 in Han Chinese based on the HapMap database. For the rest 61 SNPs, as for multiple SNPs with strong linkage disequilibrium (LD) (r^2^ ≥ 0.8) in the same region, those frequently reported or residing in a functional region were selected in priority. As a result, 48 SNPs from 34 genes were selected. As 9 SNPs (i.e. rs7756992, rs6931514, rs4712524, rs4712523 in *CDKAL1*, rs9472138 in *VEGFA*, rs1111875, rs7923837, rs5015480 in *HHEX*, rs8050136 in *FTO*) had been reported in our previous studies[28–30], the other 39 SNPs from 30 genes were included in this study (S1 Table).

In the discovery stage, 39 SNPs of 1200 type 2 diabetes cases and 1200 controls were genotyped using the TaqMan OpenArray Genotyping System (LifeTechnologies, Carlsbad, USA). DNA samples with standardized concentration were loaded and amplified on 48-sample arrays according to the manufacturer’s manual. In every chip, the equal amounts of cases and controls and two no template controls (NTCs) were simultaneously detected. The overall call rate was 98.7%. In the replication stage, 10 SNPs with the *P* value less than 0.05 in the discovery stage were further genotyped with the iPLEX Sequenom MassARRAY platform (Sequenom, Inc). For quality control, there were two NTCs in each 384-sample plate and genotyping was blindly conducted. The overall call rate of this stage was 99.5%, with the call rate > 99.0% for each SNP. The concordance rate calculated based on 150 duplicate samples for each SNP was 100%.

### Statistical analyses

Hardy-Weinberg equilibrium was assessed using a likelihood ratio test. Student’s *t* test was used to compare mean values of clinical characteristics between cases and controls. Unconditional logistic regression analysis was used to examine the association between each SNP and type 2 diabetes risks with adjustment for gender, age, and body mass index (BMI) as covariates under the additive genetic model. Conditional regression analyses on each SNP were conducted by using logistic regression with adjustment for age, sex, BMI and any of the other SNPs from the same gene under the additive genetic model.

Two methods were used to calculate the genetic score. One method treated each risk allele equally and combined them based on the count of risk alleles (each SNP was coded as 0, 1, and 2 for the number of risk alleles carried). Another method assessed the effects of the SNPs using a risk score analysis with a linear combination of the SNP genotypes weighted by their individual OR. The former was used for description and association while the latter was used for prediction. We reported the prediction value in terms of both the area under the receiver operating characteristic curve (AUC) and classification rates. Improvement in prediction was assessed after adding the weighted genetic scores to the environmental risk factors. All the statistical analyses were performed using the PLINK 1.07 and Stata software (version 11.1; StataCorp LP, College Station, Texas).

## Results

Clinical characteristics of the 6,206 participants were shown in S2 Table. There were no significant differences in the distributions of gender between cases and controls in both stages. Overall, those with type 2 diabetes were older than the controls (*P*< 0.01). The levels of BMI, FBG, triglyceride (TG), and total cholesterol (TC) were significantly higher, whereas the level of high density lipoprotein-cholesterol (HDL-C) was significantly lower in type 2 diabetes cases than they were in controls in both stages and combined analysis (*P* < 0.001). All SNPs in controls were in Hardy-Weinberg equilibrium (*P* > 0.05).

In the discovery stage with 1,200 cases and 1,200 controls, among the 39 SNPs, there were 10 SNPs from 8 genes showing suggestive associations with type 2 diabetes risk (*P* < 0.05) (Table 1). The 10 suggestive SNPs were further to be genotyped in the replication stage with an additional 1,725 cases and 2,081 controls. As shown in Table 2, 6 SNPs (rs13266634 from *SLC30A8*, rs10811661 from *CDKN2A/CDKN2B*, rs2237897, rs2237892 and rs2237895 from *KCNQ1*, rs1552224 from *CENTD2*) were consistently associated with type 2 diabetes risk, with *P* values less than 0.05. After combining the two stages together, all of the 6 SNPs were significantly associated with type 2 diabetes susceptibility after Bonferroni correction (*P* < 1.28 × 10^−3^). As 3 SNPs from *KCNQ1* showed statistically significantly associated type 2 diabetes in the study, we used logistic regression to determine the independent effects of these SNPs and found that SNP rs2237897 conferred independent risk for type 2 diabetes, as shown in S3 Table.

**Table 1:** Association between 39 SNPs from 30 loci and type 2 diabetes in the discovery stage.

**Gene region**	**SNP^a^**	**Allele^b^**	**MAF^c^ (Cases)**	**MAF^c^ (Controls)**	**OR(95%CI) ^d^**	***P*^d^**
*LOC64673/IRS1*	rs2943641	C/**T**	0.052	0.064	0.79(0.60,1.03)	0.080
*LOC64673/IRS1*	rs7578326	**A**/G	0.133	0.137	1.03(0.87,1.23)	0.713
*MRPS9/GRP45*	rs6712932	**T**/C	0.243	0.261	1.09(0.95,1.26)	0.217
*BCL11A*	rs243021	**A**/G	0.322	0.342	1.08(0.95,1.23)	0.237
*RBMS1/ITGB6*	rs7593730	C/**T**	0.162	0.151	1.09(0.92,1.29)	0.315
*IGF2BP2*	rs4402960	G/**T**	0.266	0.242	1.15(1.00,1.32)	0.055
*ADAMTS9*	rs4607103	**C**/T	0.368	0.369	1.03(0.91,1.17)	0.647
*LRTM1/WNT5A*	rs358806	C/**A**	0.182	0.180	1.00(0.86,1.18)	0.968
*ANXA5/TMEM155*	rs7659604	**C**/T	0.366	0.394	1.15(1.02,1.30)	0.026
*LOC72901/CETN3*	rs12518099	**A**/G	0.447	0.466	1.07(0.95,1.21)	0.260
*JAZF1*	rs864745	T/**C**	0.242	0.221	1.16(1.00,1.34)	0.047
*KLF14*	rs972283	**G**/A	0.305	0.313	1.05(0.91,1.20)	0.538
*SLC30A8*	rs13266634	**C**/T	0.420	0.471	1.26(1.12,1.41)	8.32×10^−5^
*TP53INP1*	rs896854	**C**/T	0.374	0.414	1.16(1.03,1.31)	0.016
*CDKN2A/CDKN2B*	rs10811661	**T**/C	0.424	0.481	1.32(1.17,1.49)	1.22×10^−5^
*CDKN2A/CDKN2B*	rs564398	T/**C**	0.118	0.119	1.01(0.83,1.22)	0.920
*CHCHD9*	rs13292136	**C**/T	0.080	0.101	1.34(1.08,1.65)	7.01×10^−3^
*PTPRD*	rs17584499	**C**/T	0.086	0.090	1.10(0.88,1.36)	0.405
*CDC123/CAMK1D*	rs10906115	**A**/G	0.354	0.372	1.07(0.94,1.22)	0.281
*CDC123/CAMK1D*	rs12779790	A/**G**	0.178	0.181	1.04(0.89,1.22)	0.612
*KCNQ1*	rs2237897	**C**/T	0.304	0.380	1.43(1.26,1.62)	3.45×10^−8^
*KCNQ1*	rs2237892	**C**/T	0.287	0.352	1.36(1.20,1.55)	2.97×10^−6^
*KCNQ1*	rs2237895	A/**C**	0.341	0.302	1.19(1.05,1.36)	7.41×10^−3^
*KCNQ1*	rs231362	**G**/A	0.107	0.116	1.11(0.92,1.35)	0.274
*CENTD2*	rs1552224	**A**/C	0.079	0.094	1.39(1.12,1.73)	2.84×10^−3^
*KCNJ11*	rs5219	C/**T**	0.407	0.390	1.09(0.96,1.24)	0.163
*KCNJ11*	rs5215	T/**C**	0.406	0.391	1.09(0.96,1.23)	0.189
*RPL9P23/API5*	rs9300039	**C**/A	0.244	0.246	1.03(0.89,1.18)	0.739
*MTNR1B*	rs1387153	C/**T**	0.421	0.425	1.02(0.90,1.15)	0.770
*TSPAN8/LGR5*	rs1495377	C/**G**	0.276	0.269	1.10(0.96,1.27)	0.156
*TSPAN8/LGR5*	rs7961581	T/**C**	0.210	0.217	1.03(0.88,1.19)	0.732
*HMGA2*	rs1531343	G/**C**	0.142	0.128	1.12(0.94,1.34)	0.190
*HIGDIC*	rs12304921	**G**/A	0.489	0.507	1.07(0.95,1.20)	0.284
*SPRY2*	rs1359790	**G**/A	0.258	0.274	1.12(0.971.28)	0.111
*C2CD4A/C2CD4B*	rs1436955	**C**/T	0.211	0.222	1.07(0.92,1.24)	0.371
*C2CD4A/C2CD4B*	rs7172432	A/**G**	0.365	0.371	1.00(0.88,1.13)	1.000
*FTO*	rs11642841	C/**A**	0.043	0.037	1.23(0.89,1.68)	0.205
*SRR*	rs391300	**C**/T	0.286	0.306	1.08(0.95,1.23)	0.248
*HNF1B*	rs4430796	A/**G**	0.308	0.298	1.07(0.93,1.21)	0.350

^a^Single nucleotide polymorphism (SNP).

^b^Major allele/minor allele. The risk alleles are in bold.

^c^Minor allele frequency (MAF).

^d^ The odds ratio (OR) with 95% confidence interval (CI) and *P* value were calculated for the risk allele in the additive genetic model by logistic regression with adjustment for age, sex and body mass index.

**Table 2:** Association between 10 suggestive SNPs and type 2 diabetes in the replication stage and combined analysis.

**Gene region**	**SNP^a^**	**Replication stage (N_case/control_ = 1,725/2,081)**				**Combined analysis (N_case/control_ = 2,925/3,281)**			
		**Case^b^**	**Control^b^**	**OR(95%CI)^c^**	***P*^c^**	**Case^b^**	**Control^b^**	**OR(95%CI)^c^**	***P*^c^**
*JAZF1*	rs864745	4.68/33.94/61.38	5.41/36.50/58.09	0.90(0.79,1.02)	0.103	5.05/35.25/59.70	5.16/35.84/58.99	1.00(0.91,1.10)	0.978
*SLC30A8*	rs13266634	15.60/50.03/34.37	18.38/49.16/32.47	1.13(1.01,1.26)	0.027	35.69/46.25/18.05	31.64/47.75/20.61	1.19(1.10,1.29)	1.43×10^−5^
*TP53INP1*	rs896854	12.51/43.16/44.33	9.84/42.24/47.93	0.88(0.79,0.98)	0.025	13.70/43.47/42.83	13.34/42.65/44.02	0.99(0.91,1.07)	0.801
*CDKN2A/CDKN2B*	rs10811661	19.11/50.73/30.16	23.78/50.55/25.66	1.30(1.16,1.44)	2.41×10^−6^	18.04/51.17/30.79	23.68/50.04/26.28	1.30(1.20,1.41)	1.34×10^−10^
*CHCHD9*	rs13292136	0.99/17.94/81.06	0.96/16.09/82.95	0.93(0.78,1.11)	0.411	0.83/16.69/82.48	1.13/16.51/82.35	1.07(0.94,1.23)	0.302
*KCNQ1*	rs2237897	8.70/41.36/49.94	12.94/46.55/40.51	1.40(1.25,1.57)	7.00×10^−9^	9.20/41.20/49.60	13.82/46.08/40.10	1.41(1.30,1.54)	9.91×10^−16^
*KCNQ1*	rs2237892	7.95/40.15/51.90	11.53/43.87/44.59	1.35(1.20,1.51)	4.92×10^−7^	8.07/40.50/51.43	12.17/43.88/43.94	1.35(1.24,1.47)	9.20×10^−12^
*KCNQ1*	rs2237895	13.53/44.76/41.71	10.23/43.15/46.62	1.25(1.12,1.40)	7.11×10^−5^	12.91/44.51/42.58	9.88/42.66/47.45	1.22(1.12,1.33)	3.29×10^−6^
*CENTD2*	rs1552224	0.58/14.38/85.04	0.82/14.80/84.39	1.19(0.98,1.44)	0.086	0.66/14.32/85.02	0.80/15.70/83.50	1.28(1.10,1.48)	9.88×10^−4^
*ANXA5/TMEM155*	rs7659604	15.14/46.99/37.87	14.39/48.01/37.60	1.04(0.93,1.15)	0.531	14.81/45.99/39.20	14.92/47.70/37.38	1.08(1.00,1.17)	0.053

^a^Single nucleotide polymorphism (SNP).

^b^Frequency of minor homozygote/heterozygote/major homozygote.

^c^The odds ratio (OR) with 95% confidence interval (CI) and P value were calculated for the risk allele indicated in Table 1 in the additive genetic model by logistic regression with adjustment for age, sex and body mass index.

**Figure 1:**
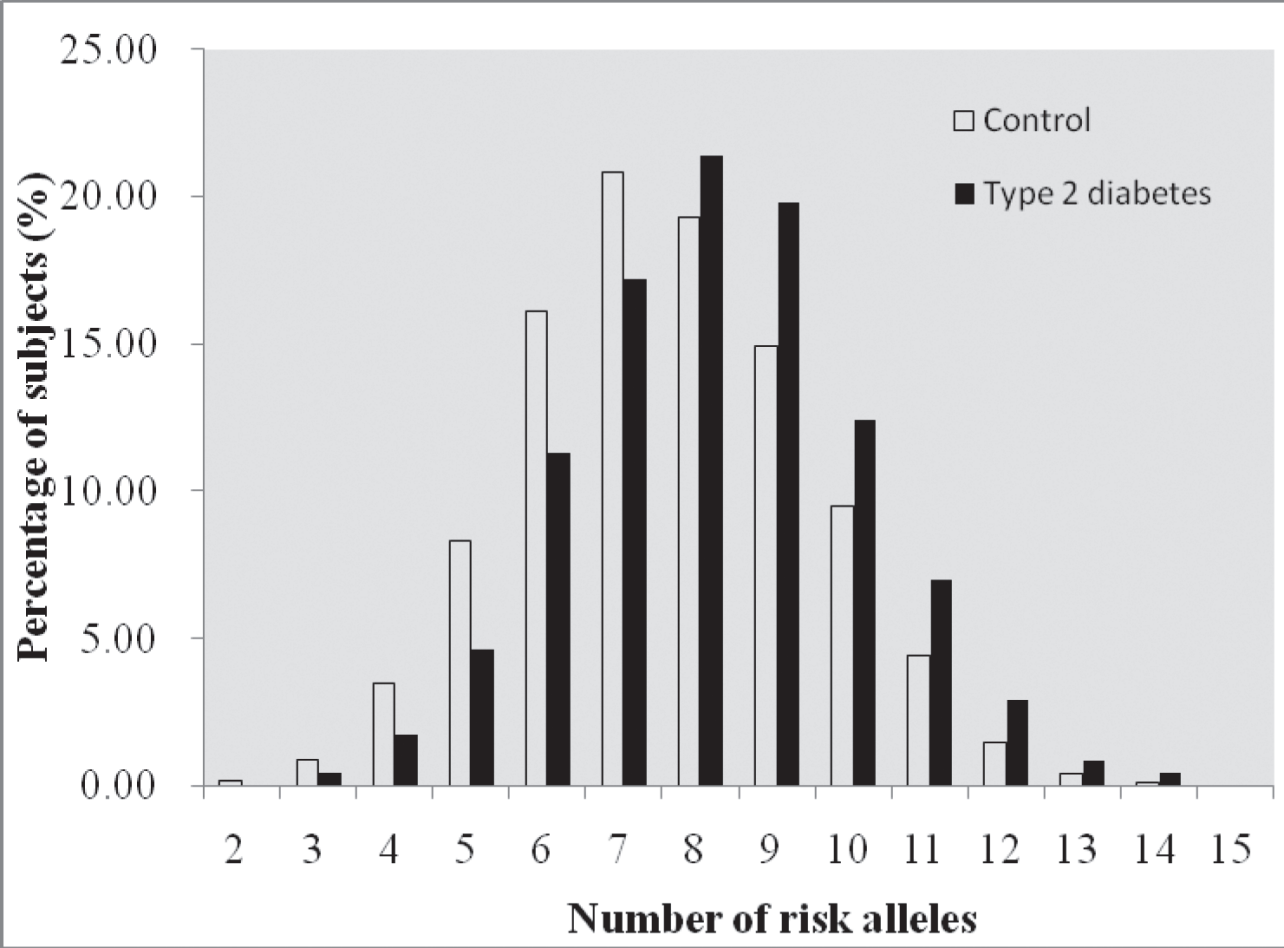
Distributions of number of risk alleles among patients with type 2 diabetes and controls with normal fasting blood glucose. Black bars signified type 2 diabetes patients, while white bars signified controls. The proportion of type 2 diabetes patients increased in the subgroups with more risk alleles.

**Figure 2:**
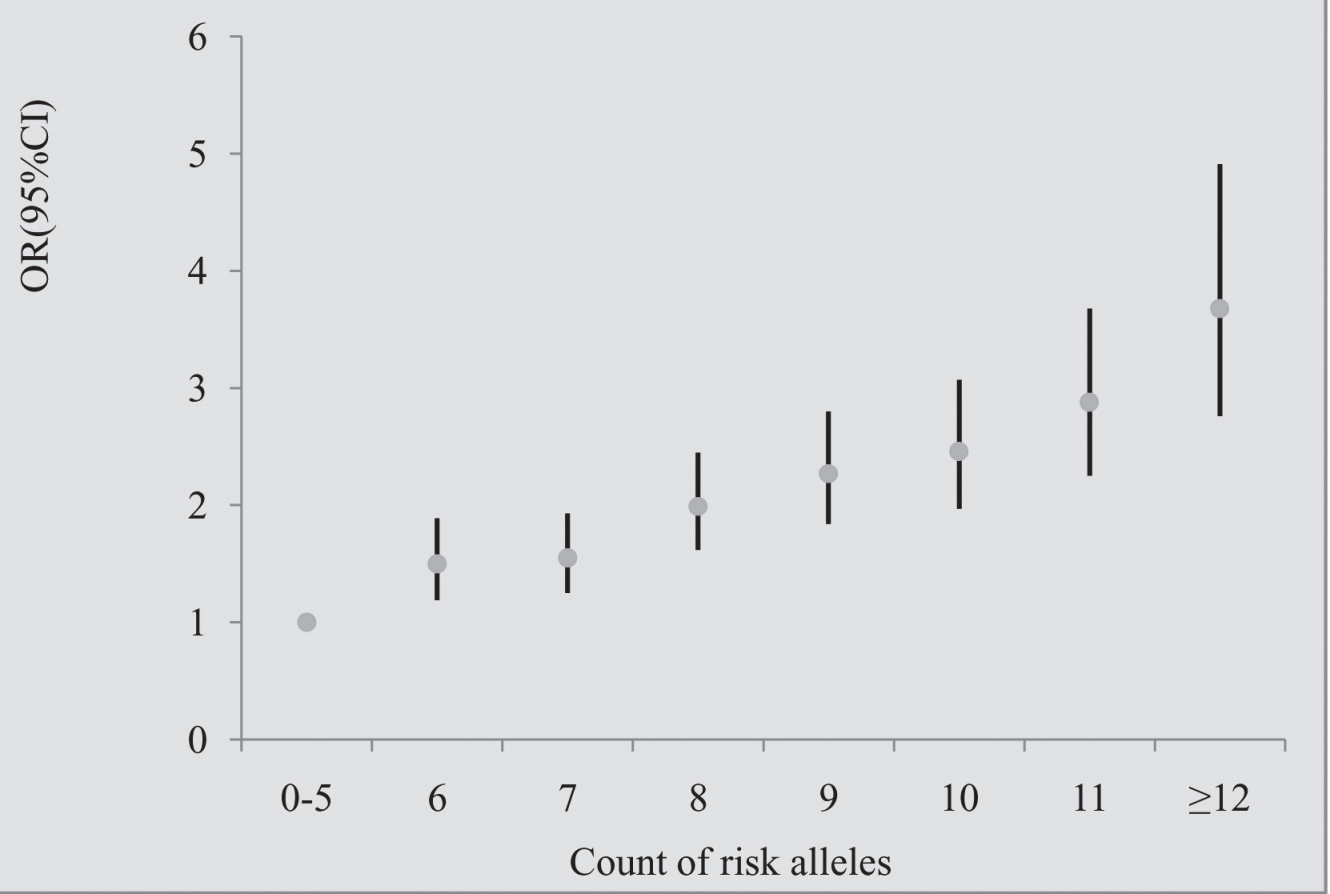
Odds ratios for risk of type 2 diabetes according to the number of risk alleles carried. As the small sample size, those carrying 0–5 risk alleles were grouped together and those carrying ≥12 risk alleles were likewise done. The combined effect of 9 single nucleotide polymorphisms was analyzed using logistic regression adjusted for age, gender and body mass index. The risk for type 2 diabetes increased with the increased number of risk alleles (*P*_trend_ = 2.0 × 10^−30^).

To investigate if genes affected type 2 diabetes additively, we examined the joint effect of risk alleles of SNPs. We included the above-mentioned 4 SNPs from 4 genes (i.e. rs13266634 from *SLC30A8*, rs10811661 from *CDKN2A/CDKN2B*, rs2237897 from *KCNQ1*, rs1552224 from *CENTD2)* and the 5 additional SNPs (i.e. rs7756992 from *CDKAL1*, rs9472138 from *VEGFA*, rs1111875, rs7923837 from *HHEX*, rs8050136 in *FTO*) that we previously reported [28–30]. All these SNPs showed independently significant association with type 2 diabetes. Thus, a total of 9 SNPs were included to analyze the cumulative effect of genetic factors on type 2 diabetes. A genotype score ranging from 0 to 18 was constructed on the basis of the number of risk alleles for each participant who had the genotyping information of the 9 SNPs. The mean (± SD) genotype score was 8.22 (± 1.88) among 2,853 subjects with type 2 diabetes and 7.58 (± 1.90) among 3,210 controls (*P* = 0.000). The proportions of type 2 diabetes cases and controls grouped by the number of risk alleles that they carried were shown in Fig. 1 and S4 Table. The percentages of individuals with type 2 diabetes increased in the subgroups with more risk alleles. With the increasing number of risk alleles, the risk of developing type 2 diabetes increased. Individuals carrying 12 or more risk alleles of the 9 SNPs (6.80% of type 2 diabetes cases and 3.64% of controls) had a nearly 4-fold increased risk for developing type 2 diabetes compared with the reference group of 0–5 risk alleles (6.76% of type 2 diabetes cases and 12.99% of controls) (Fig. 2 and S4 Table). Subjects in the upper quartile of risk score were associated with a 2.22-fold increased type 2 diabetes risk compared to those having the low quartile score (adjusted OR = 2.22, 95%CI: 1.91–2.56, *P* for trend: 5.2 × 10^−31^) (S4 Table).

**Figure 3:**
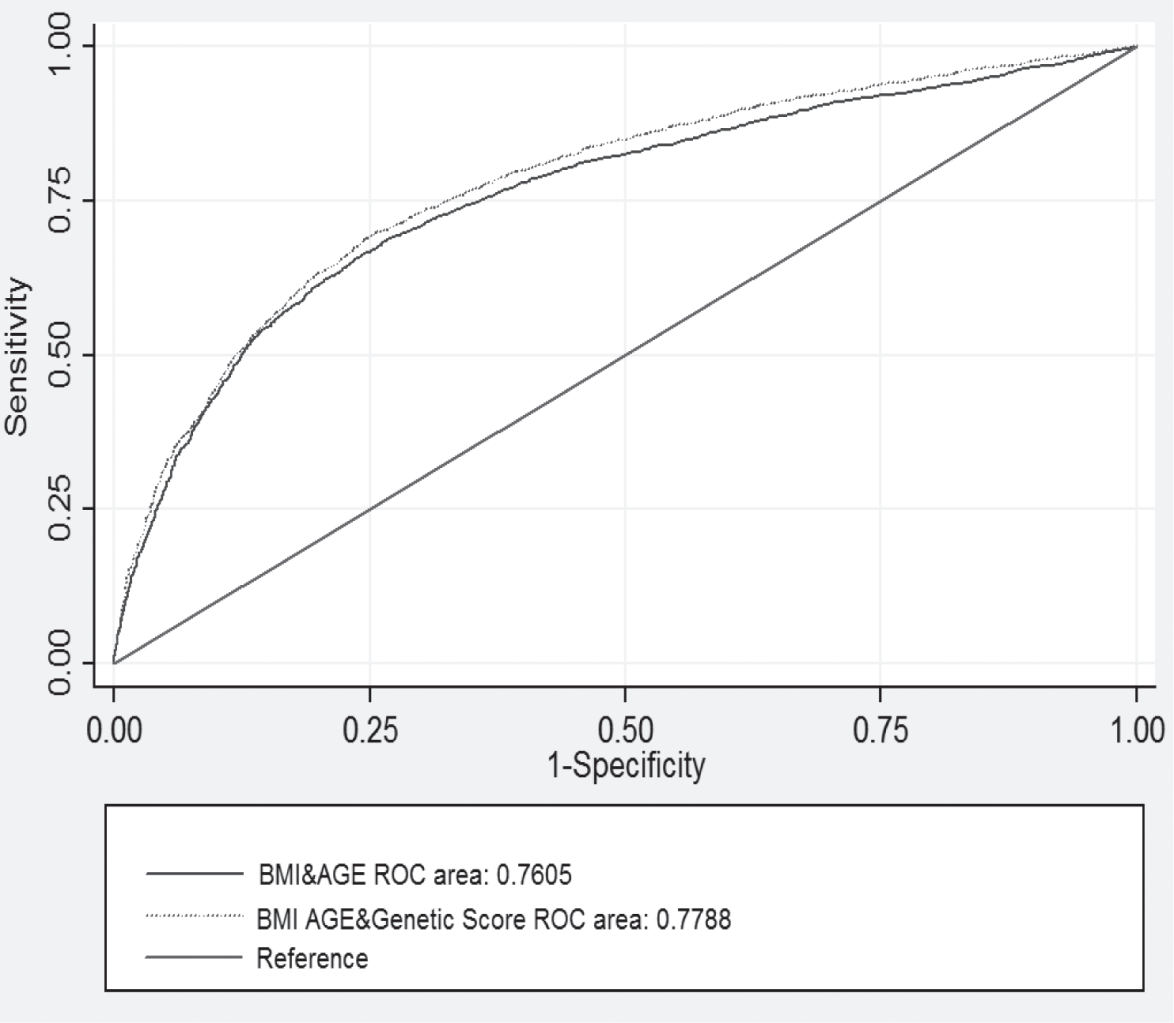
The area under the receiver operating characteristic curves for predicting type 2 diabetes based on 9 single nucleotide polymorphisms, clinical characteristics (age and body mass index). The area under the receiver operating characteristic curve (AUC) for clinical characteristics (age and body mass index) is 0.76 (95%CI: 0.75–0.78). When adding the genetic information, it improved to 0.78 (95%CI: 0.77–0.79) (*P* = 0.0000).

We evaluated the predictive value of these genetic variants in our study population contained 2,925 type 2 diabetes cases and 3281 controls. In all samples, the AUC for clinical characteristic including age and BMI was 0.76 (95%CI: 0.74–0.77) while the AUC for the weighted genetic score based on 9 SNPs was 0.60 (95%CI: 0.58–0.62). As we added the weighted genetic score to the regression model of age and BMI, the AUC slightly increased to 0.78 (95%CI: 0.77–0.79). However, the difference was statistically significant (*P* < 0.0001) (Fig. 3). The correctly classified rates were 71.07% and 72.01% before and after adding the weighted genetic score to the clinical model of age and BMI, respectively (S5 Table).

## Discussion

In the present study, we conducted a two-stage case-control study with a total of 6,206 Han Chinese subjects in order to investigate the associations of 39 SNPs from 30 genes previously identified through GWAS for type 2 diabetes. We confirmed the associations of genetic variations in *SLC30A8, CDKN2A/CDKN2B, KCNQ1, CENTD2* with type 2 diabetes. The cumulative effect analysis of 9 SNPs showed that a crude estimate of up to 3.68-fold increased the risk of type 2 diabetes in subjects carrying 12 or more risk alleles compared with those carrying 5 or fewer risk alleles. These genetic variants also showed potential utility in risk prediction for type 2 diabetes.

Our study is the first to confirm the associations between genetic variants in *CENTD2* and type 2 diabetes susceptibility in Han Chinese. *CENTD2* (also known as *ARAP1*) was initially identified as significantly associated with type 2 diabetes susceptibility, and its risk allele was associated with reduced insulin beta-cell function in nondiabetic subjects, in populations of European descent in 2010 [18]. However, two subsequent studies conducted with Han Chinese lean individuals and in the Japanese failed to replicate the association [33, 34]. Our study confirmed the association of rs1552224 in *CENTD2* with type 2 diabetes in a relatively large study of Chinese. Strawbridge, *et al* reported that the polymorphism of *CENTD2* was associated with fasting proinsulin levels in 10,701 nondiabetic adults of European ancestry and the proinsulin-raising allele was associated with a lower fasting glucose, improved β-cell function, and lower risk of type 2 diabetes [35]. *CENTD2* was significantly associated with increased plasma glucose values and decreased glucose-stimulated insulin release, suggesting that the diabetogenic effect of this locus is mediated through an impaired pancreatic beta cell function [36].

Among the confirmed SNPs in our study, the strongest association with type 2 diabetes was observed for SNP rs2237897 from the *KCNQ1*gene, as each risk allele increased the odds of type 2 diabetes by 41%. Another SNP, rs2237892, which was in moderate LD with rs2237897 in the Chinese population (r^2^ = 0.67) showed similar effect size. SNP rs2237895 (r^2^ of 0.24 with rs2237897) also showed a significant but relatively weak association. The associations of these SNPs with type 2 diabetes were identified from GWAS in Japanese or Chinese [11, 12, 15] and have been replicated in Korean [37], Pakistani [38], Indian [39], Scandinavian [40] and Chinese [22, 27, 41, 42, 43] populations. The polymorphisms were also found to be associated with increasing fasting glucose and impairment of insulin, according to the homeostasis model assessment of beta-cell function [11, 40, 41]. Based on all of this evidence, *KCNQ1* seems to have been robustly confirmed as a type 2 diabetes susceptibility gene in Han Chinese and may confer type 2 diabetes risk by impaired beta-cell function. In addition, the current study also replicated the significant associations of two other genes (*CDKN2A/CDKN2B* and *SLC30A8*) with type 2 diabetes susceptibility, which was consistent with the observations from other studies in Han Chinese [20–27].

One potential application of identifying genetic variants associated with type 2 diabetes is using genetic information to help predict risk of the disease, which may facilitate preventive interventions on those at the highest risk of type 2 diabetes. A few studies on predicting type 2 diabetes based on genetic polymorphisms have been conducted. Weedon et al. reported the AUC for 3 polymorphisms was 0.58 [44], while Vaxillaire et al. showed that the AUCs for a combination of 3 SNPs were 0.55 or 0.56 [45]. Van Hoek et al. reported the AUC was 0.58 (0.56–0.61) when including the 9 significantly associated genetic variants, and the difference between the AUCs for clinical characteristics (age, sex, and BMI), with and without the genetic polymorphisms, was significant (*P* < 0.0001) [46]. Hu evaluated the predictive value of 11 SNPs in a Chinese population and the result showed the AUC for the number of risk alleles was 0.621 (95%CI: 0.604–0.639) [21]. Our study showed the AUC of 9 SNPs was 0.60 and the difference between the AUCs for clinical characteristics (age and BMI) with and without the genetic polymorphisms was significant. These results are similar to previous reports. Despite the limited predictive values of genetic variants, as Lango et al. previously reported, patients with high genetic risk had lower BMI and earlier onset-age compared with those with relatively low genetic risk [47]. These findings highlight the potential usefulness for risk prediction of type 2 diabetes, especially in non-obese, young-onset case subjects.

The strengths of our study included the large size of the study population, the two-stage case-control design, and the relatively systematic study strategy for type 2 diabetes-related genetic variants. Despite these advantages, we did not include those established type 2 diabetes-associated genes with larger effect size in our study, such as *TCF7L2*. The discriminative accuracy of predictive genetic testing in complex diseases depends on the number of genes involved, the risk allele frequencies, and the size of the associated risks. Despite the efficient discovery of disease-associated SNPs, the case-control study is suboptimal for evaluating predictive performance. Further studies should aim at genotyping more genetic susceptibility variants and predicting new cases in prospective studies.

In conclusion, in addition to 4 genes reported previously, we further confirmed that 4 of 30 genes were associated with risk of type 2 diabetes in Han Chinese. These genes may improve the accuracy of risk prediction in combination with clinical characteristics.

## Supporting Information

S1 Table Associations between 39 SNPs from 30 loci and type 2 diabetes selected from previous GWAS. (DOC)

S2 Table Distributions of clinical characteristics of 6,206 subjects. (DOC)

S3 Table Conditional regression analysis and LD values between SNPs from *KCNQ1*. (DOC)

S4 Table Cumulative effect of associated 9 SNPs on type 2 diabetes in combined analysis. (DOC)

S5 Table Comparison of prediction of type 2 diabetes with and without weighted genetic score using classification rate. (DOC)
