# Supplementary material for: Cumulative Effect and Predictive Value of Genetic Variants Associated with Type 2 Diabetes in Han Chinese: A Case-Control Study
Source: PLoS One. 2015 Jan 14;10(1):e0116537. doi: 10.1371/journal.pone.0116537 (PMC4294637; doi:10.1371/journal.pone.0116537)
Supplement: S1 Table — (DOC) [file pone.0116537.s001.doc]

**Table S1. Associations between 39 SNPs from 30 loci and type 2 diabetes selected from previous GWAS**

| **Chr** | **Gene region** | **SNP** | **Population** | **MAF** | **Number**  **(case/control)** | **OR(95%CI)** | ***P*** | **Ref** |
| --- | --- | --- | --- | --- | --- | --- | --- | --- |
| 2 | *LOC64673/IRS1* | rs2943641 | French | 0.089 | 6258/7793 | 1.19(1.13,1.25) | 9×10^-12^ | 14 |
| 2 | *LOC64673/IRS1* | rs7578326 | European | 0.159 | 8130/38987 | 1.11(1.08,1.13) | 5×10^-20^ | 18 |
| 2 | *MRPS9/GRP45* | rs6712932 | White | 0.100 | 3073/3273 | 1.52(1.27,1.82) | 6×10^-6^ | 9 |
| 2 | *BCL11A* | rs243021 | European | 0.389 | 8130/38987 | 1.08(1.06,1.10) | 3×10^-15^ | 18 |
| 2 | *RBMS1/ITGB6* | rs7593730 | European | 0.167 | 10870/73735 | 1.11(1.08,1.16) | 4×10^-8^ | 17 |
| 3 | *IGF2BP2* | rs4402960 | Finland Sweden | 0.222 | 1464/1467 | 1.17(1.11,1.23) | 2×10^-9^ | 4 |
| 3 | *ADAMTS9* | rs4607103 | European | 0.422 | 4549/5579 | 1.09(1.06,1.12) | 1×10^-8^ | 10 |
| 3 | *LRTM1/WNT5A* | rs358806 | British | 0.227 | 1924/2938 | 1.16(1.03,1.33) | 3×10^-6^ | 6 |
| 4 | *ANXA5/TMEM155* | rs7659604 | British | 0.422 | 1924/2938 | 1.35(1.19,1.54) | 9×10^-6^ | 6 |
| 5 | *LOC72901/CETN3* | rs12518099 | French | 0.433 | 679/697 | 1.16(1.10,1.22) | 7×10^-7^ | 14 |
| 7 | *JAZF1* | rs864745 | European | 0.189 | 4549/5579 | 1.10(1.07,1.13) | 5×10^-14^ | 10 |
| 7 | *KLF14* | rs972283 | European | 0.333 | 8130/38987 | 1.07(1.05,1.10) | 2×10^-10^ | 18 |
| 8 | *SLC30A8* | rs13266634 | French | 0.478 | 3278/3508 | 1.18(0.69,1.67) | 6×10^-8^ | 3 |
| 8 | *TP53INP1* | rs896854 | European | 0.200 | 8130/38987 | 1.06(1.04,1.09) | 1×10^-9^ | 18 |
| 9 | *CDKN2A/CDKN2B* | rs10811661 | Finland Sweden | 0.405 | 1464/1467 | 1.20(1.12,1.28) | 5×10^-8^ | 4 |
| 9 | *CDKN2A/CDKN2B* | rs564398 | UK | 0.067 | 1924/2938 | 1.13(1.08,1.19) | 1×10^-6^ | 5 |
| 9 | *CHCHD9* | rs13292136 | European | 0.089 | 8130/38987 | 1.11(1.07,1.15) | 3×10^-8^ | 18 |
| 9 | *PTPRD* | rs17584499 | Chinese | 0.089 | 2798/2367 | 1.57(1.36,1.82) | 9×10^-10^ | 15 |
| 10 | *CDC123/CAMK1D* | rs10906115 | Chinese | 0.267 | 1019/1710 | 1.13(1.08,1.18) | 0.57 | 16 |
| 10 | *CDC123/CAMK1D* | rs12779790 | European | 0.133 | 4549/5579 | 1.11(1.07,1.14) | 1×10^-10^ | 10 |
| 11 | *KCNQ1* | rs2237897 | Japanese | 0.359 | 5118/4176 | 1.33(1.24,1.41) | 1×10^-16^ | 12 |
| 11 | *KCNQ1* | rs2237892 | Japanese | 0.367 | 187/1504 | 1.45(1.34,1.47) | 2×10^-42^ | 11 |
| 11 | *KCNQ1* | rs2237895 | Chinese | 0.349 | 2798/2367 | 1.29(1.19,1.40) | 1×10^-9^ | 15 |
| 11 | *KCNQ1* | rs231362 | European | 0.163 | 8130/38987 | 1.08(1.06,1.10) | 3×10^-13^ | 18 |
| 11 | *CENTD2* | rs1552224 | European | 0.089 | 8130/38987 | 1.14(1.11,1.17) | 1×10^-22^ | 18 |
| 11 | *KCNJ11* | rs5219 | Finland Sweden | 0.500 | 1464/1467 | 1.15(1.09,1.21) | 1×10^-7^ | 4 |
| 11 | *KCNJ11* | rs5215 | UK | 0.356 | 1924/2938 | 1.14(1.10,1.19) | 5×10^-11^ | 5 |
| 11 | *RPL9P23/API5* | rs9300039 | Finnish | 0.326 | 1161/1174 | 1.48(1.28,1.71) | 6×10^-8^ | 7 |
| 11 | *MTNR1B* | rs1387153 | European | 0.478 | 8130/38987 | 1.09(1.06,1.11) | 8×10^-15^ | 18 |
| 12 | *TSPAN8/LGR5* | rs1495377 | British | 0.256 | 1924/2938 | 1.28(1.11,1.49) | 7×10^-6^ | 6 |
| 12 | *TSPAN8/LGR5* | rs7961581 | European | 0.200 | 4549/5579 | 1.09(1.06,1.12) | 1×10^-9^ | 10 |
| 12 | *HMGA2* | rs1531343 | European | 0.122 | 8130/38987 | 1.10(1.07,1.14) | 4×10^-9^ | 18 |
| 12 | *HIGDIC* | rs12304921 | British | 0.466 | 1924/2938 | 2.50(1.53,4.09) | 7×10^-6^ | 6 |
| 13 | *SPRY2* | rs1359790 | Chinese | 0.278 | 1019/1710 | 1.15(1.10,1.20) | 0.71 | 16 |
| 15 | *C2CD4A /C2CD4B* | rs1436955 | Chinese | 0.244 | 1019/1710 | 1.13(1.08,1.19) | 0.73 | 16 |
| 15 | *C2CD4A/C2CD4B* | rs7172432 | Japanese | 0.398 | 4470/3071 | 1.11(1.08,1.14) | 9.0× 10⁻^14^ | 19 |
| 16 | *FTO* | rs11642841 | European | 0.056 | 8130/38987 | 1.13(1.08,1.18) | 3.0 × 10⁻^8^ | 18 |
| 17 | *SRR* | rs391300 | Chinese | 0.256 | 955/894 | 1.28(1.18,1.39) | 3.06×10^-9^ | 15 |
| 17 | *HNF1B* | rs4430796 | European | 0.289 | 8130/38987 | 1.14(1.08,1.20) | 2.0×10^-6^ | 18 |
