# Supplementary material for: Cumulative Effect and Predictive Value of Genetic Variants Associated with Type 2 Diabetes in Han Chinese: A Case-Control Study
Source: PLoS One. 2015 Jan 14;10(1):e0116537. doi: 10.1371/journal.pone.0116537 (PMC4294637; doi:10.1371/journal.pone.0116537)
Supplement: S2 Table — (DOC) [file pone.0116537.s002.doc]

**Table S2. Distributions of clinical characteristics of 6,206 subjects**

| **Variables** | **Discovery stage** | | **Replication stage** | | **Combined** | |
| --- | --- | --- | --- | --- | --- | --- |
|  | **Cases N=1,200(%)** | **Controls N=1,200(%)** | **Cases N=1,725(%)** | **Controls N=2,081(%)** | **Cases N=2,925(%)** | **Controls N=3,281(%)** |
| Males/Females (n) | 478/722 | 478/722 | 620/1105 | 756/1325 | 1098/1827 | 1234/2047 |
| Age (years) | 57.43±9.77 | 56.43±8.02^a^ | 58.77±10.31 | 56.66±10.81 ^a^ | 58.21±10.11 | 56.57±9.88 ^a^ |
| Body mass index (kg/m^2^) | 24.92±3.42 | 22.64±2.87^b^ | 25.13±3.55 | 21.82±2.44 ^b^ | 25.05±3.50 | 22.12±2.63 ^b^ |
| Fasting blood glucose (mmol/l) | 8.98±3.52 | 4.51±0.47 ^b^ | 9.07±3.30 | 4.53±0.56 ^b^ | 9.03±3.52 | 4.52±0.53 ^b^ |
| Triglyceride (mmol/l) | 2.55±2.46 | 1.32±0.45 ^b^ | 2.58±2.48 | 0.96±0.39 ^b^ | 2.57±2.47 | 1.09±0.45 ^b^ |
| Total cholesterol (mmol/l) | 5.39±1.62 | 4.64±0.71 ^b^ | 4.70±1.23 | 4.26±0.82 ^b^ | 4.98±1.44 | 4.39±0.80 ^b^ |
| High density lipoprotein-cholesterol (mmol/l) | 1.37±0.34 | 1.53±0.31 ^b^ | 1.51±0.55 | 1.68±0.40 ^b^ | 1.46±0.48 | 1.62±0.38 ^b^ |

Data are shown as means ± standard deviations for continuous variables and numbers for categorical variables.

^a^ *P* values were less than 0.01 compared between cases and controls.

^b^ *P* values were less than 0.001 compared between cases and controls.
