# Supplementary material for: Cumulative Effect and Predictive Value of Genetic Variants Associated with Type 2 Diabetes in Han Chinese: A Case-Control Study
Source: PLoS One. 2015 Jan 14;10(1):e0116537. doi: 10.1371/journal.pone.0116537 (PMC4294637; doi:10.1371/journal.pone.0116537)
Supplement: S3 Table — (DOC) [file pone.0116537.s003.doc]

**Table S3. Conditional regression analysis and LD values between SNPs from *KCNQ1***

| **Gene** | **Primary SNP** | **Risk allele** | **Unadjusted *P*^a^** | **Adjusted for SNPs** | **Adjusted *P*^b^** | **D'/r^2^** |
| --- | --- | --- | --- | --- | --- | --- |
| KCNQ1 | rs2237897 | C | 9.91×10^-16^ | rs2237892 | 0.000 | 0.86/0.67 |
|  |  |  |  | rs2237895 | 0.000 | 0.98/0.24 |
|  | rs2237892 | C | 9.20×10^-12^ | rs2237897 | 0.520 | -- |
|  |  |  |  | rs2237895 | 0.000 | 0.93/0.19 |
|  | rs2237895 | C | 3.29×10^-6^ | rs2237897 | 0.407 | **--** |
|  |  |  |  | rs2237892 | 0.087 | **--** |

^a^ *P* value calculated in the additive genetic model by logistic regression with adjustment for age, sex and body mass index.

^b^ *P* value for the primary SNP calculated in the additive genetic model by logistic regression with adjustment for age, sex, body mass index and one additional SNP in column “Adjusted for SNPs”.
