# Supplementary material for: Cumulative Effect and Predictive Value of Genetic Variants Associated with Type 2 Diabetes in Han Chinese: A Case-Control Study
Source: PLoS One. 2015 Jan 14;10(1):e0116537. doi: 10.1371/journal.pone.0116537 (PMC4294637; doi:10.1371/journal.pone.0116537)
Supplement: S4 Table — (DOC) [file pone.0116537.s004.doc]

**Table S4.** Cumulative effect of associated 9 SNPs on type 2 diabetes in combined analysis

| **Risk models** | **Cases (%)** | **Controls (%)** | **Adjusted OR (95%CI)** | ***P*** |
| --- | --- | --- | --- | --- |
| **Count of risk alleles** |  |  |  |  |
| 0-5^a^ | 193 (6.76) | 417 (12.99) | 1.00(ref.) | **-** |
| 6 | 267 (9.36) | 392 (12.21) | 1.50(1.19,1.89) | 6.5×10^-4^ |
| 7 | 394 (13.81) | 557(17.35) | 1.55(1.25,1.93) | 5.9×10^-5^ |
| 8 | 539 (18.89) | 593 (18.47) | 1.99(1.62,2.45) | 7.2×10^-11^ |
| 9 | 556 (19.49) | 539 (16.79) | 2.27(1.84,2.80) | 1.3×10^-14^ |
| 10 | 425 (14.90) | 377 (11.74) | 2.46(1.97,3.07) | 1.3×10^-15^ |
| 11 | 285 (9.99) | 218 (6.79) | 2.88(2.25,3.68) | 4.6×10^-17^ |
| ≥12 ^a^ | 194 (6.80) | 117 (3.64) | 3.68(2.76,4.91) | 5.6×10^-19^ |
| Trend |  |  |  | 2.0×10^-30^ |
| **Genetic risk score** |  |  |  |  |
| 0 (< Q25) | 456(15.59) | 803(24.47) | 1.00(ref.) | - |
| 1( Q25~Q50) | 580(19.83) | 803(24.47) | 1.28(1.09,1.50) | 2.0×10^-3^ |
| 2( Q50~Q75) | 806(27.56) | 810(24.69) | 1.77(1.52,2.06) | 1.5×10^-13^ |
| 3( ≥ Q75) | 1083(37.03) | 865(26.36) | 2.22(1.91,2.56) | 1.4×10^-26^ |
| Trend |  |  |  | 5.2×10^-31^ |

^a^ Grouping by 0-5 vs ≥12 alleles was based on sample size.
