# Supplementary material for: Cumulative Effect and Predictive Value of Genetic Variants Associated with Type 2 Diabetes in Han Chinese: A Case-Control Study
Source: PLoS One. 2015 Jan 14;10(1):e0116537. doi: 10.1371/journal.pone.0116537 (PMC4294637; doi:10.1371/journal.pone.0116537)
Supplement: S5 Table — (DOC) [file pone.0116537.s005.doc]

**Table S5. Comparison of prediction of type 2 diabetes with and without weighted genetic score using classification rate**

| **Classified** | **Without weighted genetic score** | | |  | **With weighted genetic score** | | |
| --- | --- | --- | --- | --- | --- | --- | --- |
|  | **Disease** | **Non-disease** | **Total** |  | **Disease** | **Non-disease** | **Total** |
| **+** | 1830 | 715 | 2545 |  | 1861 | 721 | 2582 |
| **-** | 1072 | 2560 | 3632 |  | 969 | 2486 | 3455 |
| Correctly classified | 71.07% | | |  | 72.01% | | |
